# Neuroimaging in Dementia: A Brief Review

**DOI:** 10.7759/cureus.8682

**Published:** 2020-06-18

**Authors:** Dipanjan Banerjee, Abilash Muralidharan, Abdul Rub Hakim Mohammed, Bilal Haider Malik

**Affiliations:** 1 Neuroscience, California Institute of Behavioral Neurosciences and Psychology, Fairfield, USA; 2 Geriatrics, Queen's Medical Center, Nottingham University Hospitals NHS Trust, Nottingham, GBR; 3 Internal Medicine, California Institute of Behavioral Neurosciences and Psychology, Fairfield, USA; 4 Internal Medicine, Kiruba Hospital, Coimbatore, IND

**Keywords:** neuroimaging, dementia, spect, pet, alzheimer’s disease, frontotemporal dementia, vascular dementia, dementia with lewy body

## Abstract

Dementia is a clinical syndrome that manifests itself with impairment in cognitive functions owing to various neurodegenerative etiologies causing severe disability in the older population. Although the diagnosis is largely dependent on clinical examination, biomarkers can significantly aid in early diagnosis of dementia, especially in those without any clinical evidence of neurocognitive impairment. These biomarkers can be discovered in cerebrospinal fluid (CSF) or can be assessed by neuroimaging. Our goal was to discuss and assess the role of different neuroimaging techniques in the early diagnosis of relatively common etiologies of dementia. We used PubMed as search engines to look for helpful articles; most of the sources used were peer reviewed. We discussed the utility of various neuroimaging techniques, such CT, MRI, positron emission tomography (PET) scan, and single-photon emission computed tomography (SPECT), in the diagnosis of dementia. We concluded that various modern neuroimaging techniques prove to be very helpful in early identification, diagnosis, and differentiation between subtypes. However, the actual clinical utility of these tests in terms of their cost-effectivity and availability remains to be seen. Ongoing research is required to further develop biomarkers for early identification and monitor the progression of different etiologies of dementia.

## Introduction and background

“It is a strange, sad irony that so often in the territory of a disease that robs an individual of memory, caregivers are often the forgotten” - Karen Wilder

In 2018, dementia affected 50 million people worldwide, a figure that is predicted to increase to 152 million by 2050. In 2018, the economic effect of dementia estimated about a trillion US dollars a year, and that is forecast to double by 2030 [1]. Early diagnosis is extremely important in terms of prognosis as it opens the door to a lot of possibilities for people with dementia and their families. An early diagnosis can help by initiating early treatment, which can slow down the progression of the disease yielding better therapeutic outcomes and increased quality of life [2]. For example, in Alzheimer's disease (AD), there is a preclinical stage, which is defined as a stage of the disease where the clinical features have not fully manifested yet to satisfy the criteria of mild cognitive impairment (MCI); however, there is biomarker evidence to suggest that the disease process is currently underway [3]. Biomarkers can be defined as measurable substances in the body that act as reliable predictors and indicators of a disease process. These biomarkers make it possible to identify the disease even before its clinical manifestation; hence, recently, this topic has been subjected to a lot of research as early intervention may provide better therapeutic results [2]. Neuroimaging is extremely helpful in the early diagnosis of dementia by demonstrating the presence of these biomarkers. Structural brain imaging can provide information about the shape, position, and, more importantly volume of brain tissue. Structural imaging techniques consist of MRI and CT, with MRI having much better resolution than CT. Functional brain imaging reveals how well cells in various brain regions are working by showing how actively the cells use sugar or oxygen. These most commonly include positron emission tomography (PET) and functional MRI (fMRI). Molecular brain imaging uses radiotracers to detect specific cellular or chemical changes; these include amyloid PET and single-photon emission computed tomography (SPECT) [4]. In this review, we aim to briefly assess and summarize the utility of various neuroimaging techniques to diagnose the most commonly prevalent etiologies of dementia.

## Review

Method

For our review article, we have used PubMed as our search engine and database. PRISMA (Preferred Reporting Items for Systematic Reviews and Meta-analyses) guidelines have not been followed. As inclusion criteria we selected studies that were done within the past five years specifically on humans. We excluded any studies done on other species or older than five years. A total of 50 articles were extracted, and most of the articles used were peer reviewed. Data have been collected ethically and legally. Keywords and search results have been summarized in Table 1.

**Table 1:** Keywords, database, and results SPECT: single-photon emission computed tomography; PET: positron emission tomography

Keyword	Database	Results
Neuroimaging	PubMed	42,780
Dementia	PubMed	38,726
SPECT	PubMed	5,151
Frontotemporal dementia	PubMed	2,269
PET scan	PubMed	22,319
Dementia with Lewy bodies	PubMed	1,631
Alzheimer’s disease	PubMed	26,716
Vascular dementia	PubMed	2,472

Discussion

Dementia and Its Etiologies

Dementia is a common neurodegenerative illness affecting large volumes of older populations globally, which is characterized by a functional decline of different cognitive domains. Diagnostic and Statistical Manual of Mental Disorders, 5th edition (DSM-V) has included dementia as “neurocognitive disorder (NCD)" and has classified according to its etiologies and severity (mild, moderate, or severe according to the daily limitation of activities). A few examples of etiologies include AD, vascular dementia (VaD), frontotemporal dementia (FTD), dementia due to Lewy body (DLB), and dementia due to Parkinson’s disease [5]. Out of numerous etiologies linked to neurocognitive disorders, AD is the most common one so far; other common etiologies being VaD, FTD, and DLB [6-8].

Alzheimer’s Disease

AD progression can be divided into three forms. Preclinical AD, where the individual is cognitively normal but has positive biomarkers. MCI due to AD, where there is evidence of declining cognitive functions, yet they do not satisfy the criteria of AD dementia. AD dementia, where the patients satisfy the criteria of diagnosis of NCD due to AD [3,9]. The pathogenesis in AD can be linked to the accumulation of amyloid-beta and the development of neurofibrillary tangles [10]. The process progresses over decades before the onset of dementia. These amyloid plaques can be visible in PET scan using 11C-labeled Pittsburgh Compound B (PiB) as radiotracer [11]. The deposits were found to be localized in neocortical brain regions, including the hippocampus [12,13]. These deposits can be demonstrated even in individuals with MCI due to AD and preclinical AD. However, various studies suggest that amyloid-beta deposits can be found in the elderly population who are cognitively normal, which highlights the fact that PiB-PET scan identifying the presence of amyloid deposition can be very sensitive, yet it is not specific [14,15]. 18-Fluorodeoxyglucose (FDG) PET scan in the resting state, looking at metabolic activity of the brain, is able to highlight areas of hypometabolism in temporal and parietal regions of the cortex including posterior cingulate region in patients with AD dementia which correlates well with the areas with amyloid deposition in the PiB-PET scan [16,17]. However, few studies showed PiB-PET positive, but cognitively normal individuals (suspected preclinical AD) did not demonstrate any metabolic abnormality in FDG-PET, although more research is required [18]. SPECT scan with 99mTc-hexamethylpropyleneamnine oxime, most commonly used to study cerebral perfusion, also shows bilateral temporoparietal hypoperfusion in AD patients (although can be asymmetric at times), a finding that can also be helpful in differentiating between other etiologies of dementia, such as FTD or VaD [19,20]. fMRI shows decreased neuronal activity in the medial temporal lobe in AD patients and may prove to be very useful in early diagnosis [21]. MRI with diffusion tensor imaging (DTI), which analyses white matter tracts, shows impairment of fibers connecting the hippocampus and posterior cingulate gyrus [21]. Structural scans such as MRI can identify volumetric changes in AD patients with dementia, MCI due to AD, and in preclinical AD. MRI can show increased gray matter atrophy predominantly in the hippocampus and cingulate cortex, which correlated well with the areas of amyloid deposition as seen in amyloid PET scan [21]. In summary, various neuroimaging techniques are able to demonstrate signs of disease activity in AD, including individuals with MCI due to AD and preclinical AD, which makes them valuable for early detection of AD.

Frontotemporal Dementia

FTD is a heterogeneous neurodegenerative disorder, which is also a fairly common cause of early-onset dementia and encompasses three clinical syndromes, out of which the most common ones are behavioral variant (bvFTD) and three language variants (semantic, non-fluent, and lopogenic) [5,22]. 3D T1 MRI shows volumetric changes in bvFTD primarily in the frontal and temporal lobes. Several studies have been able to attribute the changes to the prefrontal cortex, anterior temporal regions, the insula, anterior cingulate, and striatum, which is distinct from changes seen in the regions primarily affected in AD and hence helpful in differentiating between FTD and AD [23,24]. Structural imaging in patients with semantic variant shows asymmetric atrophy in the anteroinferior temporal lobe [25,26]. The atrophy is most commonly located on the left side; however, in some patients, the right side can be affected by volume loss [27,28]. The earliest changes in the form of volume loss can be localized to the inferior temporal and fusiform gyri, the temporal pole, and the parahippocampal and entorhinal cortex [25,29]. Nonfluent variants again primarily show left hemispheric volume loss in the inferior frontal gyrus, dorsolateral prefrontal cortex, superior temporal gyrus, and insula, which distinguishes it from other variants in the same spectrum [25,26,29]. In the lopogenic variant, volume loss is localized at the left temporoparietal and posterior cingulate atrophy [26,29]. In terms of amyloid PET, most of the FTD patients are revealed to be negative for Aβ deposition; however, patients with lopogenic variant may show positive findings due to their association with AD pathogenesis [30,31]. Cerebral metabolism and perfusion studies such as SPECT and 18FDG PET scan are also helpful, with PET having greater utility than SPECT [32]. PET shows areas of hypometabolism, which correlates well with areas of atrophy found in structural imaging as mentioned above [33,34]. The areas of hypometabolism in FTD as evidenced by FDG-PET scan have been summarized in Table 2.

**Table 2:** Areas of hypometabolism in FTD seen in FDG-PET bvFTD: behavioural variant of frontotemporal dementia; FTD: frontotemporal dementia; FDG-PET: fludeoxyglucose positron emission tomography

FTD variant	Areas of hypometabolism seen in FDG-PET
bvFTD	Medial prefrontal cortex and anterior temporal region [35,36].
Semantic	Asymmetrical left hemispheric involvement, primarily localized in the inferior temporal region entorhinal and perirhinal cortex [35,37].
Non-fluent	Inferior frontal gyrus (primarily left hemispheric [38].
Lopogenic	left frontotemporoparietal and posterior cingulate region [38].

A good share of patients have familial FTD due to genetic mutations [7]. Studies suggest the existence of a presymptomatic stage where volumetric changes in the gray matter of left cingulate cortex in T1-weighted imaging and white matter changes in uncinate fasciculus and genu corpus callosum in DTI-MRI can be demonstrated, which can serve as a diagnostic and prognostic biomarker [24]. To summarize, both volumetric studies and metabolic imaging provide excellent utility in terms of presymptomatic diagnosis of FTD, which helps to differentiate not only from AD but also helps in differentiating between different types of FTD.

Vascular Dementia

VaD is the second most common cause of dementia after AD [39]. It is often characterized by stepwise cognitive deterioration with intervening periods of stability and acute deterioration of symptoms. The pathogenesis can be attributed to vascular insults in large vessels or multiple small vessels over a prolonged period of time [40]. Hyperintense signal on T2-weighted and fluid-attenuated inversion recovery (FLAIR) images signifies the vascular insult seen in both small vessel and large vessel diseases. T2/FLAIR imaging can also identify microhemorrhages seen in VaD [41,42].

Dementia With Lewy Body

DLB is also a very common form of dementia and may present with neurocognitive decline, visual symptoms, and parkinsonism [43]. Volumetric studies have shown DLB patients have more preserved temporal lobe, amygdala, and hippocampal volumes compared to AD patients [44]. FDG-PET shows significantly reduced uptake in the visual cortex in DLB patients [45]. Assessing dopaminergic function carries value in DLB, which can be done by dopaminergic transporter (DAT) scan by SPECT using (123I) FP-CIT as a radiotracer. This shows markedly decreased dopaminergic activity in DLB patients [46,47]. 123I-metaiodobenzylguanidine (MIBG) cardiac scintigraphy can be helpful in distinguishing DLB from AD; however, the utility may be limited by interference in outcome by co-existing cardiac disorders and diabetes mellitus [48,49].

## Conclusions

Hence, after careful review, we conclude that neuroimaging not only establishes its crucial role in the diagnosis of various etiologies of dementia but also it is proven to be exceedingly helpful in differentiating between multiple subtypes within a particular etiology. It also plays a leading role of neuroimaging in early diagnosis of dementia, which will pave the way for early initiation of treatment, thus delaying the progression of the disease by pharmacological means leading to better patient care. Some of the more sophisticated imaging techniques, although with their proven benefits, may face limited clinical utility, taking into consideration their cost or limited availability. The future focus of neuroimaging in dementia is very likely to shift towards multimodal imaging combining various metabolic, functional, and structural imaging to diagnose the condition, predict the progression, and monitor therapeutic benefits.
